# Knockdown of Gonadotropin-Releasing Hormone II Receptor Impairs Ovulation Rate, Corpus Luteum Development, and Progesterone Production in Gilts

**DOI:** 10.3390/ani14162350

**Published:** 2024-08-14

**Authors:** Amy T. Desaulniers, Rebecca A. Cederberg, Clay A. Lents, Brett R. White

**Affiliations:** 1 Department of Animal Science, University of Nebraska-Lincoln, Lincoln, NE 68583-0908, USA; desaulniers@unl.edu (A.T.D.); rcederberg2@unl.edu (R.A.C.); 2 United States Department of Agriculture, Agricultural Research Service, U.S. Meat Animal Research Center, Clay Center, NE 68933-0166, USA; clay.lents@usda.gov

**Keywords:** GnRH-II, GnRH-II receptor, ovulation rate, *Corpus luteum*, progesterone, autocrine/paracrine regulation, gene knockdown, porcine, steroid hormone

## Abstract

**Simple Summary:**

Reproductive loss is a major constraint to efficient swine production. Our goal is to better understand the biology of reproduction in female swine. Gonadotropin-releasing hormone II and its receptor are proteins produced by the pig ovary, but their function is not fully understood. To further explore their role in female reproduction, we measured reproductive hormones in the circulating blood of genetically modified pigs that produce less gonadotropin-releasing hormone II receptor throughout the body, and their littermate control siblings during the estrous (reproductive) cycle. Additionally, we compared the morphological characteristics of the ovary and hormone production by the *Corpora lutea* (ovarian structures that produce the critical pregnancy hormone, progesterone). Ovaries from females with reduced gonadotropin-releasing hormone II receptor levels ovulated 17% fewer oocytes (eggs), contained *Corpora lutea* with smaller steroidogenic cells, containing 23% less progesterone, with 18% less progesterone into circulation compared with littermate controls. These data suggest that GnRH-II and its receptor play a role in regulating the number of oocytes ovulated, *Corpora lutea* development, and progesterone production in pigs. A better understanding of female reproduction will lead to novel interventions to enhance the reproductive efficiency of pigs, increasing the productivity and sustainability of the swine industry.

**Abstract:**

Reproduction is classically controlled by gonadotropin-releasing hormone (GnRH-I) and its receptor (GnRHR-I) within the brain. In pigs, a second form (GnRH-II) and its specific receptor (GnRHR-II) are also produced, with greater abundance in peripheral vs. central reproductive tissues. The binding of GnRH-II to GnRHR-II has been implicated in the autocrine/paracrine regulation of gonadal steroidogenesis rather than gonadotropin secretion. Blood samples were collected from transgenic gilts, with the ubiquitous knockdown of GnRHR-II (GnRHR-II KD; *n* = 8) and littermate controls (*n* = 7) at the onset of estrus (follicular) and 10 days later (luteal); serum concentrations of 16 steroid hormones were quantified by high-performance liquid chromatography tandem mass spectrometry (HPLC-MS/MS). Upon euthanasia, ovarian weight (OWT), ovulation rate (OR), and the weight of each excised *Corpus luteum* (CLWT) were recorded; HPLC-MS/MS was performed on CL homogenates. During the luteal phase, serum progesterone concentration was reduced by 18% in GnRHR-II KD versus control gilts (*p* = 0.0329). Age and weight at puberty, estrous cycle length, and OWT were similar between lines (*p* > 0.05). Interestingly, OR was reduced (*p* = 0.0123), and total CLWT tended to be reduced (*p* = 0.0958) in GnRHR-II KD compared with control females. Luteal cells in CL sections from GnRHR-II KD gilts were hypotrophic (*p* < 0.0001). Therefore, GnRH-II and its receptor may help regulate OR, CL development, and progesterone production in gilts.

## 1. Introduction

Ovarian dysfunction remains a major limitation to efficient swine production, decreasing farrowing rates and litter sizes [1]. The classical gonadotropin-releasing hormone (GnRH-I; pGlu-His-Trp-Ser-Tyr-Gly-Leu-Arg-Pro-Gly), hailed as the master regulator of reproduction, stimulates the synthesis and secretion of the gonadotropins, luteinizing hormone (LH) and follicle-stimulating hormone (FSH) [2,3,4]. Yet, a second form of GnRH (GnRH-II; pGlu-His-Trp-Ser-**His**-Gly-**Trp**-**Tyr**-Pro-Gly) has also been identified in mammals [5]. GnRH-II has been completely conserved for 500 million years of evolution, suggesting a critical biological role [6]. To date, 89 mammals maintain the GnRH-II gene [7] but it appears to be silenced in the majority of species [8]. Only 10 species [human (*Homo sapien*), rhesus macaque (*Macaca mulatta*), marmoset (*Callithrix jacchus*), tarsier (*Tarsius syrichta*), tree shrew (*Tupaia belangeri*), guinea pig (*Cavia porcellus*), musk shrew (*Suncus murinus*), common shrew (*Sorex Araneus*), horse (*Equus caballus*), and pig (*Sus scrofa*)] possess the appropriate genomic sequence to produce a functional decapeptide [8]. Unlike GnRH-I, the production of GnRH-II is ubiquitous [9], with the greatest abundance (up to a 30-fold increase) in extra-hypothalamic tissues such as the kidney, prostate, and bone marrow, suggesting a diverse role in other body systems [5,10,11,12]. The scarce production of GnRH-II in hypothalamic regions [12] does not support a role in the regulation of gonadotropin secretion. Indeed, GnRH-II is a poor stimulator of gonadotropin release in vitro [13,14,15,16] and in vivo [10,12], likely occurring via interaction with the classic GnRH receptor (GnRHR-I) [7]. GnRH-II can bind GnRHR-I, albeit with 10-fold less affinity than GnRH-I [17]. Notably, GnRH-II is produced in numerous reproductive organs in the female (e.g., ovary, uterus, endometrium, and myometrium) and the male (e.g., testis, epididymis, seminal vesicles, and prostate) [7], suggesting a non-hypothalamic role in the regulation of reproduction.

A receptor specific for GnRH-II (GnRHR-II) has also been identified in mammals [11,18]. Like GnRH-II, GnRHR-II is produced ubiquitously, including extra-pituitary reproductive tissues in the male (testis, epididymis, seminal vesicles, bulbourethral gland, and prostate) and female (ovary, *Corpus luteum*, oviduct, uterus, placenta, and mammary gland) [7]. To date, the gene for GnRHR-II has been identified in the genomes of 83 species [7]. However, most species cannot produce a functional GnRHR-II due to the presence of gene coding errors or deletions [17,19,20,21,22]. Only eight species [orangutan (*Pongo pygmaeus*), African green monkey (*Ceropithecus aethiops*), rhesus macaque (*Macaca mulatta*), marmoset (*Callithrix jacchus*), tree shrew (*Tupaia belangeri*), kangaroo rat (*Dipodomys Ordii*), pig (*Sus scrofa*), and African elephant (*Loxodonta Africana*)] possess the appropriate gene sequence to produce a functional receptor for GnRH-II [8].

The biological role of GnRH-II and GnRHR-II in mammals has remained elusive since their discovery in 1998 and 2001, respectively. The pig represents one of the few species that produces both GnRH-II and GnRHR-II [8], positioning swine as a prominent model to study the function of this unique ligand–receptor complex. Previous data from our laboratory suggest that GnRH-II and its receptor promote steroidogenesis in an autocrine/paracrine manner within porcine testes [10,23,24]. To further elucidate the role of GnRH-II and its receptor, our group developed a transgenic GnRHR-II knockdown (KD) swine model using RNA interference [23]. Males have a 69% reduction in testicular GnRHR-II expression and impaired testosterone biosynthesis during pubertal development; however, LH secretion is unaffected [23]. Likewise, the serum concentration of 17β-estradiol was reduced in GnRHR-II KD boars [25]. These data support the hypothesis that GnRH-II and its receptor directly regulate testicular steroidogenesis in swine. 

Thus far, the role of GnRH-II and its receptor in the female pig has received little attention. Our group demonstrated that GnRH-II is abundant in porcine follicular fluid and GnRHR-II is expressed in the porcine CL [25]. In addition, the serum progesterone concentration was reduced in GnRHR-II gilts during the estrous cycle and gestating GnRHR-II KD sows at 42 days of pregnancy compared to controls [25]. In other species, GnRH-II and its receptor have been linked to a wide variety of functions surrounding female reproduction, such as the modulation of sexual behavior based on nutritional status [26,27,28,29,30,31], placental function [32,33,34,35], implantation [36,37,38], and the reproductive cancers of women (e.g., ovarian, breast and endometrial; [39,40,41,42,43]). Interestingly, GnRH-II and/or GnRHR-II have also been implicated in ovarian steroidogenesis, specifically the regulation of progesterone synthesis in humans [44], rhesus macaques [45], and baboons [46]. These data suggest that GnRH-II and its receptor are novel regulators of female reproduction via diverse actions from GnRH-I. Accordingly, the aim of this study was to elucidate the role of GnRH-II and its receptor in porcine ovarian function. Based on previous data from our group and others, we hypothesized that GnRHR-II KD would preferentially alter ovarian progesterone production.

## 2. Materials and Methods

### 2.1. Animals

All procedures were performed within the UNL Department of Animal Science (Lincoln, NE). Hemizygous GnRHR-II KD (*n* = 8) and non-transgenic littermate controls (*n* = 7) were produced from a total of 3 litters. This transgenic swine line was initially developed by subcloning a small hairpin RNA sequence targeting porcine GnRHR-II into a lentiviral-based vector. Thereafter, lentiviral particles were produced and microinjected (1.15 × 10^9^ IU/mL) into the perivitelline space of 1-cell embryos prior to transfer to a recipient sow [23]. One founder animal was generated and subsequently bred to develop the GnRHR-II KD line [23]. GnRHR-II KD pigs maintain a transgene that is stability-integrated on chromosome 14 [23]. The transgene contains two ubiquitous promotors; the cytomegalovirus promoter drives the production of the reporter gene ZsGreen1 and the human U6 promotor drives the production of short hairpin RNA (shRNA) specific to porcine *GnRHR-II* [23]. Genotyping was performed as described previously [47]. Piglets were nursed ad libitum by their dam until approximately 21 d of age, when they were weaned and group housed with ad libitum access to feed and water. At approximately 185 days of age and market weight (165 kg), pigs were limit fed approximately 2.5 kg of feed daily. All diets were formulated to meet National Research Council guidelines for each stage of production [48].

### 2.2. Growth, Pubertal Development, and Cyclicity

**Figure 1:**
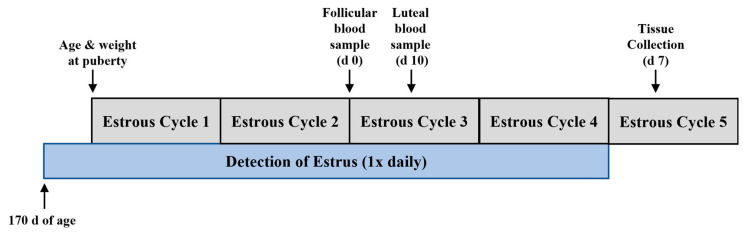
Experimental design. The once daily detection of estrus began at approximately 170 d of age. Puberty was considered the first display of behavioral estrus. The detection of estrus continued for a total of five consecutive estrous cycles. At the onset of the third estrous cycle (d 0), blood was collected via jugular venipuncture (follicular sample) and 10 d later (luteal sample). Animals were euthanized and reproductive tissues were collected on approximately day 7 of the fifth estrous cycle.

GnRHR-II KD (*n* = 8) and littermate control (*n* = 7) gilts were weighed at birth, weaning, during development (40, 60, 80, 100, 125, 145 and 165 d of age), and at puberty. Beginning at approximately 170 d of age, gilts were exposed to a mature boar, and behavioral signs of estrus (e.g., lordosis) were assessed by trained technicians once daily (Figure 1). Puberty was considered the first display of behavioral estrus. The detection of estrus continued for a total of five consecutive estrous cycles (Figure 1). The length of each estrous cycle was recorded.

### 2.3. Blood Collection

On the third estrous cycle, blood was collected via jugular venipuncture at the onset of estrus (day 0; follicular sample) and 10 d later (luteal sample; Figure 1) from GnRHR-II KD (*n* = 8) and littermate control (*n* = 7) gilts. Blood was allowed to clot at 4 °C before serum was isolated and stored at −20 °C until use.

### 2.4. Tissue Collection

GnRHR-II KD (*n* = 8) and littermate control (*n* = 7) gilts were euthanized according to recommendations from the American Veterinary Medical Association [49] approximately 7 d after onset of their fifth behavioral estrus (Figure 1). This collection day was selected to examine the developing CL prior to maximal luteal tissue progesterone production (days 8–12) [50]. The ovaries were weighed, and ovulation rate (number of *Corpora lutea*) was recorded. For a subset of animals (*n* = 4 per line), each CL was excised from the ovary and weighed. The total CL weight was then calculated (sum of individual CL weights per animal). For all animals, samples of *Corpora lutea* were flash frozen (stored long term at −80 °C), preserved in RNA*later* (100 mg/mL; Qiagen, Valencia, CA; stored at −20 °C), and fixed in 4% paraformaldehyde for later laboratory analysis.

### 2.5. Digital Droplet PCR

Digital droplet PCR (ddPCR) was used to quantify the abundance of *GnRHR-II* mRNA in CL samples of transgenic (*n* = 8) and littermate control (*n* = 7) gilts, as described previously by our group [23]. The advantages of ddPCR include absolute quantification as well as high sensitivity, precision, and reproducibility [51]. Samples were removed from RNA*later* and homogenized in TRIzol (1 mL; Invitrogen, Carlsbad, CA, USA) with a Tissue Tearor (Biospec Products Inc., Bartlesville, OK, USA). Total RNA was extracted using standard laboratory procedures with chloroform and isopropyl alcohol; resultant RNA was DNAse treated, quantitated via spectrophotometer (NanoDrop Technologies, Inc., Wilmington, DE, USA) and reverse transcribed into complementary DNA (cDNA) with the iScript cDNA synthesis kit (Bio-Rad Laboratories, Richmond, CA, USA) according to the manufacturer’s instructions.

The PCR amplification of GnRHR-II was performed using forward (F-CCCCGGACAAGGAAGGG) and reverse (R-AAGGAGCGACGGAGGGTCAA) primers (Integrated DNA Technologies, Coralville, IA, USA) as well as a FAM-labeled TaqMan MGB probe (ATGATGCCCCTGCCGG; Applied Biosystems, Foster City, CA, USA) as previously reported by our group [23]. Each reaction contained the cDNA template (diluted 1:3), 900 nM of each primer, 250 nM of the probe and 1X ddPCR Supermix for Probes (#186-3024; Bio-Rad). Droplets were generated using the QX200 Droplet Generator (Bio-Rad) according to the manufacturer’s instructions. A C1000 Touch Thermal Cycler (Bio-Rad, Richmond, CA, USA) was utilized with the following conditions: 95 °C for 10 min (enzyme activation), 94 °C for 30 s (denaturation) followed by 60 °C for 1 min (annealing/extension; 40 cycles), and 98 °C for 10 min (enzyme deactivation). Droplets were read via the QX200 droplet reader (Bio-Rad) and analyzed using QuantaSoft Software 1.7 (Bio-Rad). The housekeeping gene utilized was ACTB; each reaction contained the cDNA template (diluted 1:30), 100 nM of each primer (F-AACTCCATCATGAAGTGCGACG; R-GATCCACATCTGCTGGAAGG) and 1X EvaGreen ddPCR Supermix (#186-4034; Bio-Rad). Cycling conditions for ACTB were as follows: 95 °C for 5 min (enzyme activation), 95 °C for 30 s (denaturation) followed by 60 °C for 1 min (annealing/extension; 40 cycles), 4 °C for 5 min and 90 °C for 5 min (signal stabilization).

### 2.6. Histological Analysis

Samples from GnRHR-II KD (*n* = 7) and littermate control (*n* = 6) gilts were fixed in 4% paraformaldehyde overnight at 4 °C. After fixation, CL samples were stored in 70% ethanol until further processing. Samples were prepared by dehydrating with 100% ethanol, clearing with CitriSolv (Fisher Scientific; Pittsburgh, PA, USA), paraffin embedding, sectioning (7 µm) and mounting sections on UltraStick slides (Gold Seal Products, Portsmouth, NH, USA). Slides were then deparaffinized with CitriSolv and rehydrated with decreasing dilutions of ethanol; sections were then stained with hematoxylin (Fisher Scientific) and eosin (VWR Scientific, Bridgeport, NJ, USA). Slides were viewed via an Olympus BX51 Microscope (Olympus America Inc.; Center Valley, PA, USA), and images were captured using CellSens digital imaging software 1.3 (Olympus America Inc.) and an Olympus DP71 camera. Slides were imaged at 400× magnification and the luteal cell area was determined via CellSens digital imaging software (Olympus America Inc.). The area of an individual luteal cell (µm^2^) was measured by tracing the plasma membrane using the closed polygonal tool in CellSens. A total of eighty cells were measured per animal from two random regions of the CL section. Each cell was measured twice, and the values were averaged.

### 2.7. Mass Spectrometry

Serum and CL samples were subjected to high-performance liquid chromatography tandem mass spectrometry (HPLC/MS-MS) at Biocrates Life Sciences AG (Innsbruck, Austria) using the AbsoluteIDQ Stero17 Panel as previously described [52,53,54,55]. Serum samples were analyzed from all animals [GnRHR-II KD (*n* = 8) and littermate control (*n* = 7) gilts] while CL samples were analyzed from a subset of animals [GnRHR-II KD (*n* = 4) and littermate control (*n* = 4) gilts]. Serum samples were prepared via solid phase extraction as previously described [52]. CL samples were homogenized at Biocrates Life Sciences AG as follows. After weighing, samples were transferred into 15 mL Precellys tubes (Bertin Technologies, Montigny le Bretonneux, France) and suspended in 3 µL ethanol/phosphate extraction buffer (85%/15%; volume/volume) per mg tissue. Ceramic beads (1 g of 1.4 mm beads and 1 g of 2.8 mm beads) were added, and samples were then sonicated, vortexed, and homogenized using a Precellys-24 instrument (Bertin Technologies). Homogenization occurred at 5800 rpm (3 × 30 s with 30 s pauses) at 2 °C. The extracts were centrifuged in a Rotana-460 centrifuge at 1500× *g* for 2 min and the supernatant was isolated. Supernatant aliquots (250 µL) were diluted 1:1 with water and then utilized for mass spectrometric measurement. For measuring progesterone, an aliquot of the supernatant was diluted 1:10,000 with extraction buffer and then 250 µL of the diluted supernatant was diluted 1:1 with water for mass spectrometry. Absolute hormone concentrations were determined for 17 different steroid hormones simultaneously from each sample. Analytes included androgens [(androstenedione, dehydroepiandrosterone (DHEA), DHEA sulfate, dihydrotestosterone, testosterone, etiocholanolone, and androsterone)], estrogens (estrone and 17β-estradiol), progestogens (17α-hydroxyprogesterone and progesterone), mineralocorticoids (aldosterone, corticosterone, 11-deoxycorticosterone) and glucocorticoids (cortisol, 11-deoxycortisol, and cortisone). The specificity and sensitivity of each analyte has been previously reported [52]. Serum progesterone concentration obtained from HPLC/MS-MS were validated in house by radioimmunoassay (MP Biomedicals, Santa Ana, CA, USA; Cat. #07-170102) according to the manufacturer’s instructions and as previously described in the pig [56].

### 2.8. Statistical Analysis

Data were subjected to analysis of variance (ANOVA) via the MIXED procedure of the Statistical Analysis System (SAS; version 9.4; Cary, NC, USA). Normality was assessed (Shapiro–Wilk test) prior to data analysis; some data (e.g., hormone concentrations) were transformed (e.g., log) as needed before statistical analysis to meet normality assumptions. However, all transformed data were back-transformed to the original scale for presentation and interpretation. The statistical model for most metrics solely included line (transgenic versus control) as the fixed effect. For the serum hormone data, phase (follicular or luteal) and line by phase interaction were also included in the model as fixed effects. The interaction was removed from the model if not significant, and therefore only the main effects of either line or phase are presented when significant. For growth data, age was used as the repeated measure, with animal as the subject. The degrees of freedom for the pooled error term were calculated using the Satterthwaite approximation. Based on the Akaike’s information criterion, a heterogeneous autoregressive function with lag equal to 1 was used to model the covariance structure for the repeated measures. For all analyses, animal was the experimental unit, litter was a random effect, and a *p*-value ≤ 0.05 was considered significant, whereas a *p*-value of ≤0.10 and ≥0.06 was considered a tendency. Results are presented as least squares means (LSMEANS) ± the standard error of the mean (SEM).

## 3. Results

### 3.1. Growth, Pubertal Development, and Estrous Cyclicity

**Figure 2:**
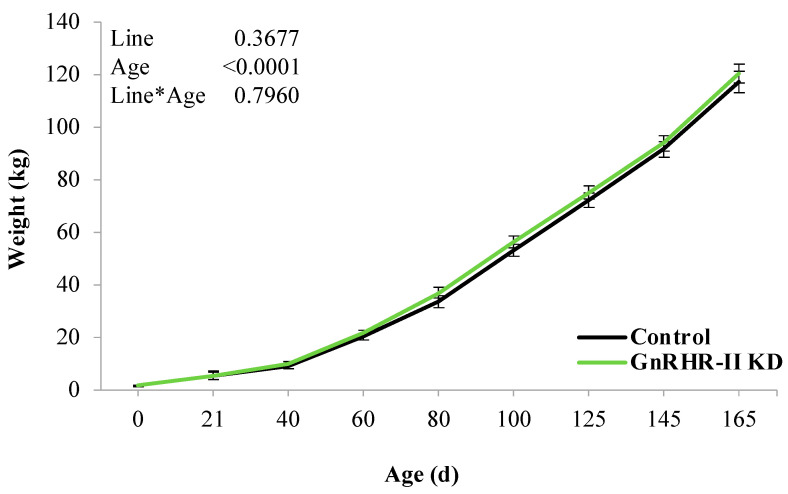
Body weights were not different between GnRHR-II KD (*n* = 8) and littermate control (*n* = 7) gilts over time. Body weight was recorded at birth, weaning, and during pre-pubertal development (40, 60, 80, 100, 125, 145 and 165 d of age). Results are presented as least squares means (LSMEANS) ± the standard error of the mean (SEM). Line, *p* = 0.3677; Age, *p* < 0.0001; Line × Age, *p* = 0.7960.

**Figure 3:**
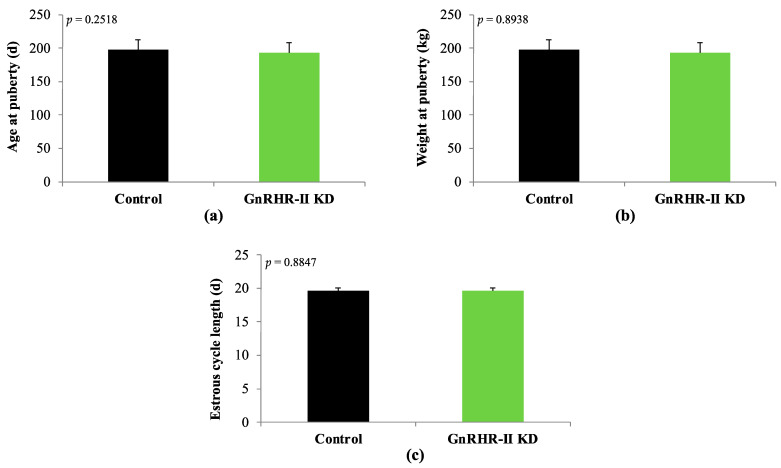
Age at puberty (**a**), weight at puberty (**b**), and estrous cycle length (**c**) in GnRHR-II KD (*n* = 8) and littermate control (*n* = 7) gilts. No line effects were detected (*p* > 0.10). Results are presented as least squares means (LSMEANS) ± the standard error of the mean (SEM).

As expected, an effect of age was detected for body weight (*p* < 0.0001). However, there was no effect of line (*p* = 0.3677) or a line by age interaction (*p* = 0.7960; Figure 2). GnRHR-II KD and control gilts had similar body weights during development (Figure 2). Likewise, weight at puberty did not differ between lines (*p* = 0.8938; Figure 3). Age at puberty and estrous cycle length was also similar between lines (*p* > 0.10; Figure 3). 

### 3.2. Corticosteroids in Circulation

**Figure 4:**
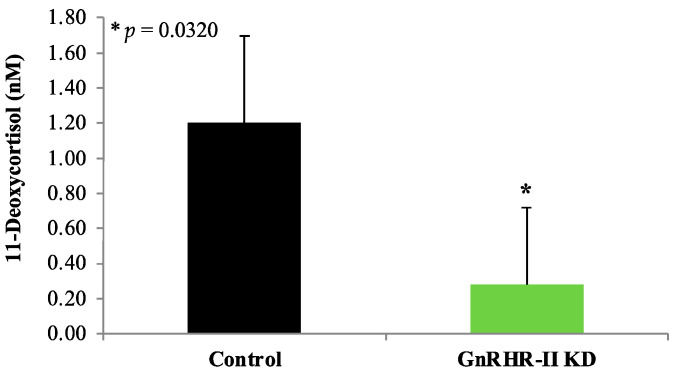
Concentrations of 11-deoxycortisol in blood serum samples from GnRHR-II KD (*n* = 8) and littermate control (*n* = 7) gilts during the follicular and luteal phases of the estrous cycle. There was no effect of phase or line by phase interaction (*p* > 0.05). However, there was an overall effect (*p* = 0.0320) of line; GnRHR-II KD gilts had reduced circulating concentrations. Results are presented as least squares means (LSMEANS) ± the standard error of the mean (SEM). * *p* < 0.05.

Line by phase interactions were not detected for any corticosteroids (*p* > 0.10). An effect of phase (*p* < 0.05) was detected for the following corticosteroids: 11-deoxycortisol, corticosterone, cortisol, and cortisone (Table 1). Serum concentrations for these hormones were greater during the follicular compared with luteal phase (*p* < 0.05; Table 1). An effect of line was detected for 11-deoxycortisol (*p* = 0.0320), with a lower circulating concentration present in GnRHR-II KD compared to littermate control gilts (Figure 4). Aldosterone concentration was not affected by line, phase, or line by phase interaction (*p* > 0.10). Etiocholanolone was not detectable in any gilt serum samples.

### 3.3. Progestogens in Circulation

**Figure 5:**
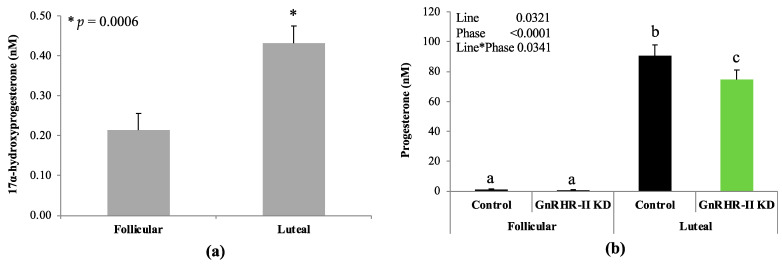
Progestogen concentrations in blood serum samples from GnRHR-II KD (*n* = 8) and littermate control (*n* = 7) gilts during the follicular and luteal phase of the estrous cycle. Neither an effect of line (GnRHR-II KD versus control) nor a line by phase interaction was detected for 17α-hydroxyprogesterone, so these data are not reported. However, a phase effect (*p* = 0.0006) was detected for 17α-hydroxyprogesterone, with the concentration greater during the luteal phase (**a**). A line by phase interaction (*p* = 0.0341) was detected for progesterone; GnRHR-II KD gilts produced less progesterone during the luteal phase (**b**). Results are presented as least squares means (LSMEANS) ± the standard error of the mean (SEM). ^a,b,c^ Divergent letters differ significantly (*p* < 0.05); * *p* < 0.05.

No effect of line or line by phase interaction was observed for 17α-hydroxyprogesterone (*p* > 0.10). An effect of phase was detected for 17α-hydroxyprogesterone (*p* = 0.0006; Figure 5a). As expected, luteal samples (0.43 ± 0.04 nM) contained 2-fold more 17α-hydroxyprogesterone compared to follicular samples (0.21 ± 0.04 nM; Figure 5a; *p* = 0.0006). A line by phase interaction was detected for progesterone (*p* = 0.0341; Figure 5b), indicating that GnRHR-II knockdown affected progesterone concentration during only one sampling period (luteal phase). Serum progesterone concentration was not different between genotypes during the follicular phase (*p* > 0.10; Figure 5). However, serum progesterone concentration increased significantly in both lines during the luteal phase, as expected (*p* < 0.05; Figure 5b). Interestingly, however, GnRHR-II KD gilts had 18% less progesterone in circulation during the luteal phase (74.7 ± 6.5 nM) compared with littermate control females (90.6 ± 7.0 nM; *p* < 0.05; Figure 5b). 

### 3.4. Androgens in Circulation

**Figure 6:**
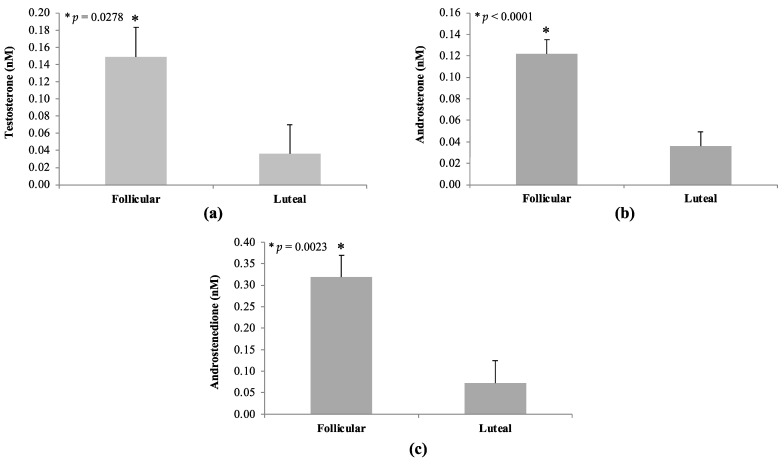
Androgen concentrations during the follicular phase and luteal phase in blood serum samples from GnRHR-II KD (*n* = 8) and littermate control (*n* = 7) gilts. No effect of line (GnRHR-II KD versus control) nor line by phase interaction was detected for any androgen examined (*p* > 0.05); therefore, these data are not reported. A phase effect (*p* < 0.05) was detected for testosterone (**a**), androsterone (**b**) and androstenedione (**c**) with concentrations greater during the follicular phase. Results are presented as least squares means (LSMEANS) ± the standard error of the mean (SEM). * *p* < 0.05.

Line by phase interactions were not observed (*p* > 0.10) for any androgens examined. There was also no effect of line on serum androgen concentrations (*p* > 0.05). However, phase effects were detected for testosterone, androsterone, and androstenedione (*p* < 0.05; Figure 6). Concentrations of these steroids were greater during the follicular versus luteal phase in gilts. Serum testosterone concentration was 4-fold greater in follicular (0.15 ± 0.03 nM) compared with luteal (0.04 ± 0.03 nM) samples (*p* = 0.0278; Figure 6). Androsterone was 3-fold greater in follicular (0.12 ± 0.01 nM) versus luteal (0.04 ± 0.01 nM) serum samples (*p* < 0.0001; Figure 6). The concentration of androstenedione was 5-fold greater in follicular (0.32 ± 0.05 nM) compared to luteal (0.07 ± 0.05 nM) samples (*p* = 0.0023; Figure 6). The following androgens were either completely or largely undetectable in all gilt serum samples: etiocholanolone, DHEA, DHEA sulfate, and dihydrotestosterone.

### 3.5. Estrogens in Circulation

**Figure 7:**
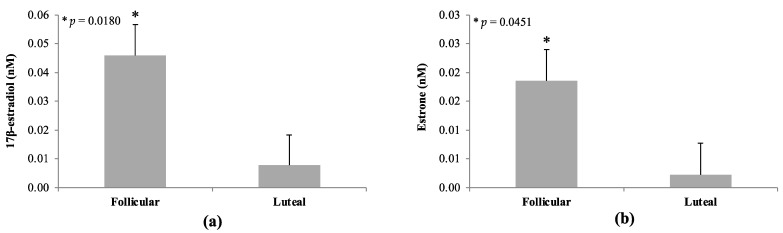
Estrogen concentrations during the follicular and luteal phase in blood serum samples from GnRHR-II KD (*n* = 8) and littermate control (*n* = 7) gilts. No effect of line (GnRHR-II KD versus control) nor line by phase interaction was detected for estrogens (*p* > 0.05); therefore, these data are not reported. A phase effect (*p* < 0.05) was detected for both 17β-estradiol (**a**) and estrone (**b**) with concentrations greater during the follicular phase compared with luteal phase. Results are presented as least squares means (LSMEANS) ± the standard error of the mean (SEM). * *p* < 0.05.

A line by phase interaction was not detected (*p* > 0.10) for the two estrogens (estrone and 17β-estradiol) examined. Likewise, there was no effect of line on serum estrogen concentrations (*p* > 0.10). As expected, there was an effect of phase on serum estrogen concentrations, with estrogen levels elevated during the follicular phase (*p* < 0.05; Figure 7). The concentration of 17β-estradiol was increased 10-fold in samples collected during the follicular (0.05 ± 0.01 nM) versus luteal (0.008 ± 0.01 nM) phase (*p* = 0.0180; Figure 7). Likewise, estrone concentration was increased by 10-fold in follicular (0.02 ± 0.005 nM) versus luteal (0.002 ± 0.005 nM) serum samples (*p* = 0.0451; Figure 7).

### 3.6. Corpus luteum Metrics and GnRHR-II Quantification

**Figure 8:**
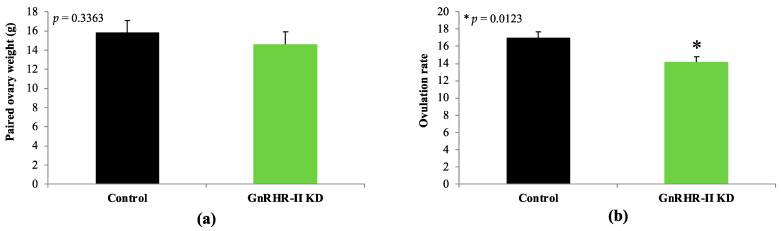
Ovarian characteristics in GnRHR-II KD (*n* = 8) and littermate control (*n* = 7) gilts. Paired ovary weight (**a**) was similar (*p* > 0.10) between lines, but ovulation rate (number of *Corpora lutea*) (**b**) was reduced (*p* = 0.0123) in GnRHR-II KD gilts compared with littermate controls. Results are presented as least squares means (LSMEANS) ± the standard error of the mean (SEM). * *p* < 0.05.

**Figure 9:**
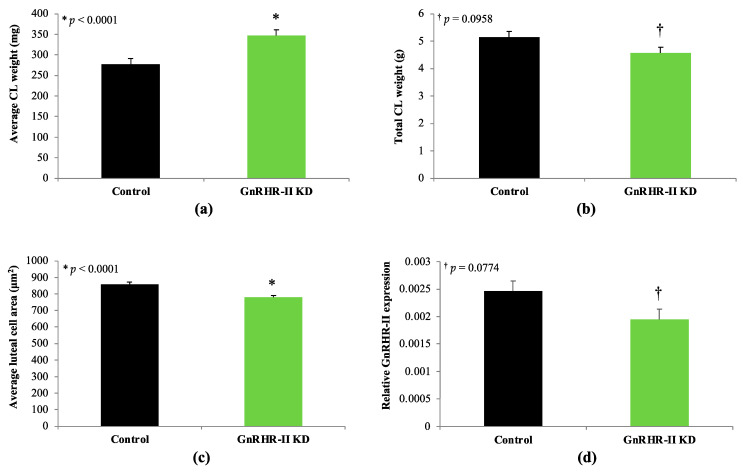
*Corpus luteum* (CL) metrics and GnRHR-II expression in CL samples from GnRHR-II KD and littermate control gilts. Average individual CL weight was greater (*p* < 0.0001) in GnRHR-II KD (*n* = 4) versus control (*n* = 4) gilts (**a**). Total CL weight per ovary tended to be reduced (*p* = 0.0958) in GnRHR-II KD (*n* = 4) versus control (*n* = 4) gilts (**b**). Luteal cell area was reduced (*p* < 0.0001) in GnRHR-II KD (*n* = 7) versus littermate control (*n* = 6) gilts (**c**). Expression of GnRHR-II tended to be reduced (*p* = 0.0774) by 21% in CL from GnRHR-II KD (*n* = 8) versus control (*n* = 7) gilts (**d**). Results are presented as least squares means (LSMEANS) ± the standard error of the mean (SEM). * *p* < 0.05; † *p* < 0.10.

Paired ovary weight did not differ between GnRHR-II KD and littermate control gilts (*p* = 0.3363; Figure 8). However, ovulation rate (number of *Corpora lutea*) was decreased by 17% in GnRHR-II KD gilts (14.1 ± 0.7) versus littermate controls (17.0 ± 0.7; *p* = 0.0123; Figure 8). The weight of each CL was 20% greater in transgenic compared to littermate control females (346.7 ± 14.3 mg versus 277.1 ± 13.7 mg, respectively; *p* < 0.0001; Figure 9). Total CL weight, however, tended to be reduced by 10% in GnRHR-II KD versus control females (4.6 ± 0.2 g versus 5.1 ± 0.2 g, respectively; *p* = 0.0958; Figure 9). Histological analysis revealed luteal cell hypotrophy in CL samples from GnRHR-II KD gilts; luteal cell area was reduced by 9% in CL sections from GnRHR-II KD compared with littermate control gilts (778.0 ± 11.8 µm^2^ versus 858.5 ± 12.7 µm^2^; *p* < 0.0001; Figure 9; Supplementary Figure S1). Expression of GnRHR-II tended to be reduced by 21% in CL samples from transgenic gilts compared to littermate controls (*p* = 0.0774; Figure 9).

### 3.7. Steroids within the Corpus luteum

The concentrations of corticosteroids did not differ in CL samples between GnRHR-II KD and littermate control gilts (*p* > 0.10; Table 2). For progestogens, line differences were detected for progesterone but not 17α-hydroxyprogesterone (Table 3). The progesterone concentration tended to be reduced by 23% in CL from GnRHR-II KD compared with littermate control females (128,849 ± 13,355 fmol/mg versus 166,838 ± 13,355 fmol/mg, respectively; *p* = 0.0910; Table 3). For estrogens, line differences were revealed for estrone but not 17β-estradiol (Table 3). Estrone tended to be reduced by 50% in CL samples from GnRHR-II KD gilts compared with littermate controls (0.9 ± 0.3 fmol/mg tissue versus 1.8 ± 0.3 fmol/mg tissue, respectively; *p* = 0.0914; Table 3). In contrast, 17β-estradiol was unaffected by line (*p* > 0.10; Table 3). The concentrations of androgens (DHEA sulfate, androstenedione, testosterone, androsterone) in CL samples were not different between genotypes (*p* > 0.10; Table 3). The following steroids were undetectable in CL homogenates: aldosterone, DHEA, and DHT.

## 4. Discussion

In the present study, steroids from four classes (estrogens, androgens, progestogens and corticosteroids) were examined in serum during the estrous cycle (follicular or luteal phase) as well as in CL homogenates. Notably, GnRHR-II KD preferentially affected the concentration of ovarian steroids in serum and/or CL homogenates (e.g., progestogens, estrogens). In contrast, adrenal steroid hormones (e.g., corticosteroids) were largely unaffected by GnRHR-II KD. In marmosets, another species that produces both GnRH-II and GnRHR-II [8], GnRHR-II is lowly expressed in the adrenal gland [11] but has not been localized to a specific cell type. Expression of GnRHR-II has not been examined in the adrenal gland of swine. If present, however, GnRHR-II expression would presumably be reduced in GnRHR-II KD gilts due to the ubiquitous nature of the U6 promotor, which drives the production of shRNA-targeting porcine GnRHR-II in these animals [23]. We observed an overall effect of line for serum 11-deoxycortisol concentration, which was reduced in GnRHR-II KD gilts. This weak corticosteroid is a precursor to cortisol, which was not different between lines. Thus, it seems unlikely that this finding equates to a substantial biological impact. Instead, global GnRHR-II KD appears to have more pronounced effect on ovarian function.

Previously, our group detected GnRH-II within the follicular fluid of pre-ovulatory porcine follicles [25]. In support of this, 17β-estradiol stimulates GnRH-II expression in granulosa-luteal cells [57] and granulosa cells secrete both 17β-estradiol [58] and GnRH-II [46]. GnRH-II expression in granulosa cells is robust in pre-ovulatory follicles, whereas it is not detectable in early follicles (e.g., primordial, primary, secondary) [59]. In contrast, theca cells of the pre-ovulatory follicle were only weakly positive for GnRH-II immunostaining [59]. These data suggest that GnRH-II and 17β-estradiol may interact in an autocrine/paracrine manner within the ovarian follicle. Therefore, we postulated whether serum 17β-estradiol concentration would differ between GnRHR-II KD and littermate control gilts during the follicular phase of the estrous cycle. However, we were unable to detect any differences in circulating 17β-estradiol or estrone concentrations between lines. Due to the experimental design of the current study, we did not collect blood samples during peak estradiol production (which occurs prior to the onset of estrus in swine [60]). Thus, we are unable to rule out the possibility that peak 17β-estradiol secretion may differ between GnRHR-II KD and control females. Follow-up studies in gilts fitted with indwelling jugular catheters are currently underway to fully characterize the endocrine profiles of GnRHR-II KD gilts.

GnRHR-II KD preferentially impaired the metrics of CL development and function. For instance, serum progesterone concentration (during the luteal phase) was reduced in GnRHR-II KD gilts. Consistent with this, serum progesterone concentration quantified via radioimmunoassay at day 10 of four consecutive estrous cycles in a subset of animals was also reduced in GnRHR-II KD gilts (*n* = 4) compared to littermate controls (*n* = 3; unpublished data). Notably, ovulation rate (indicated by CL number) was also significantly reduced in GnRHR-II KD (14.1) versus control (17.0) gilts. Therefore, the 18% reduction in the circulating progesterone concentration in GnRHR-II KD females might be attributed to 17% fewer CL than littermate control gilts. Moreover, total CL weight was reduced in GnRHR-II KD gilts, and we detected a tendency for a lower progesterone concentration (~23%) in CL homogenates from GnRHR-II KD compared with control females. Luteal cells of GnRHR-II KD gilts were hypotrophic, suggesting impaired steroidogenic capacity. Conversely, a reduced average luteal cell area could also indicate a greater proportion of small luteal cells (which produce less progesterone) versus large luteal cells [61]. Thus, the cause of the significantly reduced progesterone in the circulation of transgenic gilts during the luteal phase appears to be due to a significant reduction in ovulation rate (number of *Corpora lutea*) as well as a tendency for a reduction in total CL mass and progesterone production per mg of CL tissue. 

GnRH-II has been implicated in the regulation of luteal cell function in humans and primates [44,45,46]. For example, GnRH-II was detected in both large luteal cells and immortalized granulosa-luteal cells of women [59]. Large luteal cells are derived from granulosa cells and produce an elevated basal concentration of progesterone [61]. Notably, GnRH-II was not identifiable in small luteal cells [59], which express LH receptors and produce progesterone in response to LH [61]. The treatment of granulosa-luteal cells with human chorionic gonadotropin (hCG), an agonist of LH, enhanced GnRH-II expression [44], whereas GnRH-II treatment inhibited both basal and hCG-stimulated progesterone secretion from human granulosa-luteal cells [44]. The primary cultures of human granulosa-luteal cells treated with RU486 (progesterone receptor antagonist) increased *GnRH-II* expression in a time- and dose-dependent fashion [57]. Interestingly, granulosa cells secreted GnRH-II [46] and a culture of human granulosa-luteal cells for a 10 d increased *GnRH-II* mRNA expression, suggesting an autocrine/paracrine positive feedback loop [57]. Further, the treatment of human granulosa-luteal cells with 17β-estradiol resulted in a dose-dependent increase in *GnRH-II* mRNA expression [57]. Therefore, the expression of *GnRH-II* in the ovary appears to be regulated in both an endocrine and autocrine/paracrine manner by several different reproductive hormones, including 17β-estradiol, progesterone, LH/hCG, and GnRH-II itself. 

Kang et al. [44] demonstrated that GnRH-II impairs progesterone production from human luteal cells. Likewise, low doses of GnRH-II (2, 4 or 8 µg/day) potently inhibited secretion of progesterone from rhesus monkeys during days 3–11 of the luteal phase, whereas elevated GnRH-II doses (16 or 32 µg/day) had no effect [45]. In the baboon, exogenous GnRH-II administration suppressed the production of progesterone from cultured granulosa cells by 75% [46]. Binding kinetics suggested that these effects were mediated by GnRHR-II within the baboon ovary [46]. Collectively, these data are compelling and suggest that GnRH-II and its receptor play an underappreciated role in modulating *Corpus luteum* function. Regardless of the mechanism, our results suggest that progesterone production from the CL is impaired by the KD of GnRHR-II in gilts. 

In the present study, we detected a tendency for a modest reduction (21%) in GnRHR-II expression in the CL of transgenic gilts. Variable knockdown has also been noted in other U6-shRNA swine models [62]. Interestingly, the magnitude of GnRHR-II KD in transgenic CL (21%) corresponded to a modest reduction in CL number (17%) and progesterone production in both luteal serum samples (18%) and CL tissue (23%) of transgenic gilts. Furthermore, GnRHR-II KD females had a suppressed (24%) serum progesterone concentration during early pregnancy (d 42) compared with littermate control animals [25]. Although reduced, the concentration of progesterone was apparently still sufficient to maintain pregnancy, as we did not have any issues generating transgenic offspring. 

In addition to exploring the role of GnRH-II and its receptor in the reproductive physiology of the pig, this work also provides new information about steroidogenesis during the porcine estrous cycle. As expected, we detected a phase of the estrous cycle effect (follicular versus luteal) for ovarian steroid hormone concentrations. Estrogens and androgens were greater in serum during the follicular phase, whereas progestogens were elevated in blood samples during the luteal phase. Interestingly, however, we also detected phase effects on lesser studied corticosteroids (e.g., 11-deoxycortisol, corticosterone, cortisone) and androgens (e.g., androsterone). These hormones were elevated during the follicular phase compared with the luteal phase, which is the first report in swine to our knowledge. Likewise, these data reveal that androgens, estrogens, and corticosteroids are also products of the porcine CL, although in dramatically lower abundance compared with progesterone. The primary corticosteroid, androgen, and estrogen made by the porcine CL are cortisone, androstenedione, and estrone, respectively. The biological significance of this discovery requires further elucidation. 

## 5. Conclusions

GnRH-II is an ancient peptide whose biological role has been elusive, largely due to a lack of animal models. This study is the first to report the function of GnRHR-II in the female pig. GnRHR-II KD gilts have a reduced ovulation rate (number of *Corpora lutea*), altered *Corpus luteum* development, and reduced progesterone production. These swine represent the first genetically engineered animal model to study the function of GnRH-II and its receptor in mammals, and are currently being utilized to identify the molecular mechanisms linking GnRHR-II KD with ovarian function in female pigs. Both ovulation rate and progesterone production are essential for female fertility; thus, GnRH-II and its receptor represent novel molecular targets to enhance reproductive efficiency in swine. Given the recent discovery of genes for GnRH-II and GnRHR-II in numerous mammalian species [7], this novel system is expected to be biologically relevant and unexplored in a wide range of animals.

## Figures and Tables

**Table 1:** Corticosteroid concentrations in blood serum from GnRHR-II KD (*n* = 8) and littermate control (*n* = 7) gilts during the follicular and luteal phases of the estrous cycle.

Hormone (nM) #	Follicular	Luteal	SEM	*p*-Value
11-deoxycortisol	1.3	0.2	0.5	0.0320
Corticosterone	3.4	1.4	0.7	0.0103
Cortisol	56.2	28.9	9.1	0.0084
Cortisone	16.2	9.1	2.7	0.0081

# No effect of line (GnRHR-II KD versus control) or line by phase interaction was detected for the indicated corticosteroids (*p* > 0.05); therefore, these data are not reported. However, a phase effect (*p* < 0.05) was detected for 11-deoxycortisol, corticosterone, cortisol, and cortisone, with the concentrations of all steroids greater during the follicular phase compared with luteal phase. Results are presented as least squares means (LSMEANS) ± the standard error of the mean (SEM).

**Table 2:** Corticosteroid concentrations within *Corpus luteum* homogenates from GnRHR-II KD (*n* = 4) and littermate control (*n* = 4) gilts.

Hormone (fmol/mg Tissue) #	Control	GnRHR-II KD	SEM	*p*-Value
11-Deoxycorticosterone	15.6	13.4	1.9	0.4569
11-Deoxycortisol	2.8	2.4	0.8	0.7100
Corticosterone	3.4	2.9	1.4	0.7991
Cortisol	6.6	6.7	2.3	0.9789
Cortisone	88.8	70.4	26.9	0.6452

# No differences were detected between lines (*p* > 0.10). Results are presented as least squares means (LSMEANS) ± the standard error of the mean (SEM).

**Table 3:** Progestogens, androgens, and estrogens concentrations within *Corpus luteum* homogenates from GnRHR-II KD (*n* = 4) and littermate control (*n* = 4) gilts.

Hormone (fmol/mg Tissue) #	Control	GnRHR-II KD	SEM	*p*-Value
17α-Hydroxyprogesterone	169.1	79.4	48.9	0.2425
Progesterone	166,838	128,849	13,355	0.0910
Dehydroepiandrosterone sulfate	19.9	23.7	7.5	0.7328
Androstenedione	69.4	38.2	19.9	0.3104
Testosterone	3.6	3.3	1.3	0.8751
Androsterone	1.0	1.1	0.3	0.7165
Estrone	1.8	0.9	0.3	0.0914
17β-estradiol	0.9	1.1	0.3	0.7630

# A tendency for an effect of line was detected for progesterone and estrone (*p* < 0.10); GnRHR-II KD gilts tended to produce less estrone and progesterone per mg of *Corpus luteum* tissue. Results are presented as least squares means (LSMEANS) ± the standard error of the mean (SEM).

## Data Availability

The data presented in this study are available upon request from the corresponding author.

## Supplementary Materials

The following supporting information can be downloaded at: https://www.mdpi.com/article/10.3390/ani14162350/s1, Figure S1: Representative histological images (40×) of Corpus luteum samples from GnRHR-II knockdown (Panel A; *n* = 7) and littermate control (Panel B; *n* = 6) gilts collected at approximately day 7 of the fifth estrous cycle.
