# Supplementary material for: Knockdown of Gonadotropin-Releasing Hormone II Receptor Impairs Ovulation Rate, Corpus Luteum Development, and Progesterone Production in Gilts
Source: Animals (Basel). 2024 Aug 14;14(16):2350. doi: 10.3390/ani14162350 (PMC11350859; doi:10.3390/ani14162350)
Supplement: Supplementary file 1 [file animals-14-02350-s001.zip › animals-3098052-supplementary.pptx]

## Slide 1
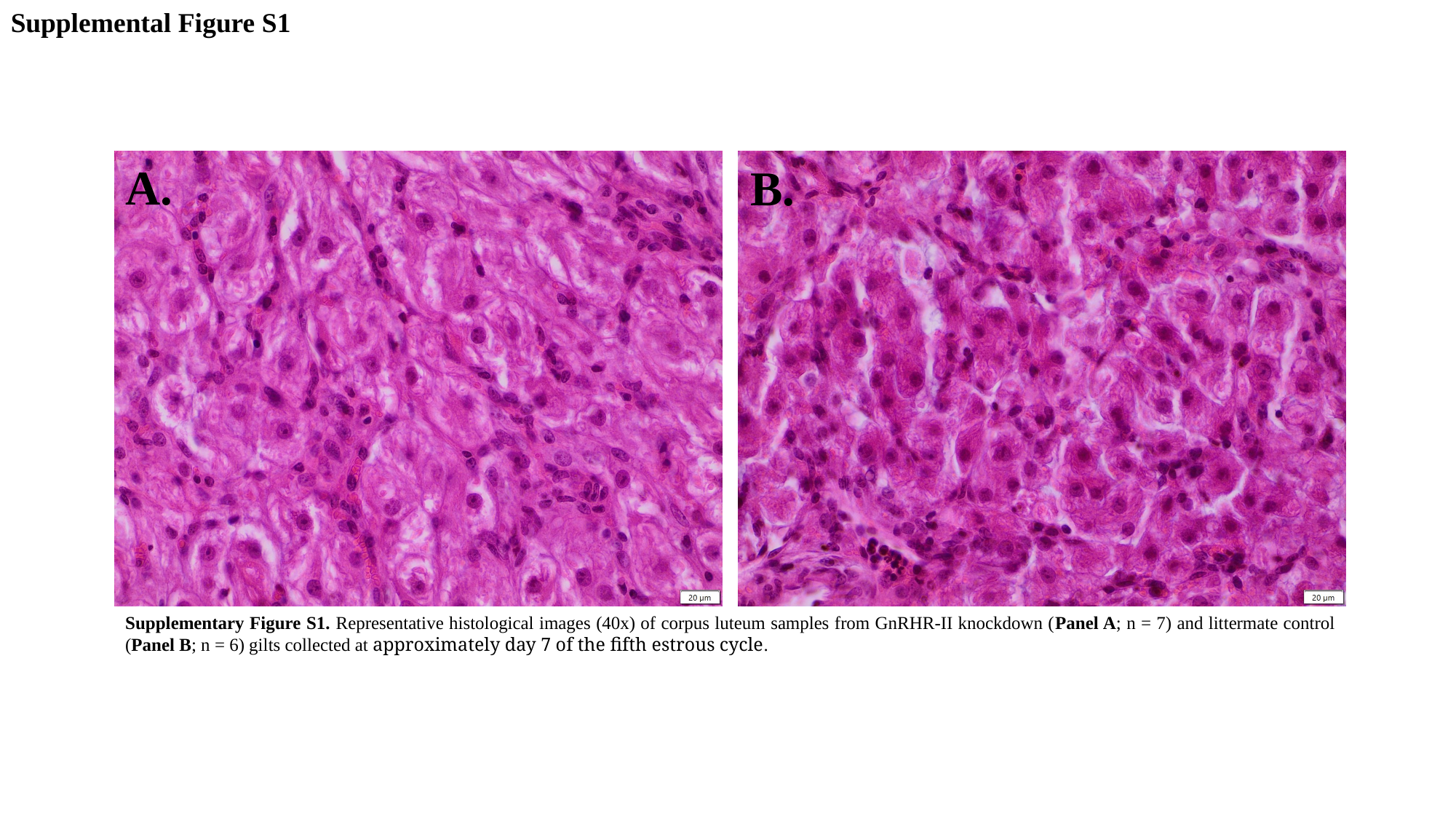

Supplemental Figure S1
A.
B.
Supplementary Figure S1. Representative histological images (40x) of corpus luteum samples from GnRHR-II knockdown (Panel A; n = 7) and littermate control (Panel B; n = 6) gilts collected at approximately day 7 of the fifth estrous cycle.
